# A Cancer Health Needs Assessment Reveals Important Differences Between US-Born and Foreign-Born Latinos in California

**DOI:** 10.3389/fonc.2022.883200

**Published:** 2022-07-07

**Authors:** Juanita Elizabeth Quino, Fabian Perez, Angelica Perez, April Pangia Vang, Leonie Avendano, Julie Dang, Moon S. Chen, Alexa Morales Arana, Sienna Rocha, Miriam Nuno, Primo N. Lara, Laura Fejerman, Luis G. Carvajal-Carmona

**Affiliations:** ^1^ Comprehensive Cancer Center, University of California Davis, Sacramento, CA, United States; ^2^ Genome Center, University of California Davis, Davis, CA, United States; ^3^ Department of Public Health Sciences, University of California Davis, Davis, CA, United States; ^4^ California Department of Public Health, Environmental Health Investigations Branch, Richmond, CA, United States; ^5^ Department of Surgery, University of California Davis Health, Sacramento, CA, United States; ^6^ Department of Biochemistry and Molecular Medicine, School of Medicine, University of California Davis, Davis, CA, United States; ^7^ Center for Advancing Cancer Health Equity, School of Medicine, University of California Davis, Davis, CA, United States

**Keywords:** health disparities, nativity, needs assessment, Latino health, preventative screenings

## Abstract

**Background:**

Cancer is the leading cause of death among Latinos, the largest minority population in the United States (US). To address cancer challenges experienced by Latinos, we conducted a catchment area population assessment (CAPA) using validated questions from the National Cancer Institute (NCI) population health assessment supplement at our NCI-designated cancer center in California.

**Methods:**

A mixed-methods CAPA was administered by bilingual-bicultural staff, with a focus on understanding the differences between foreign-born and US-born Latinos.

**Results:**

255 Latinos responded to the survey conducted between August 2019 and May 2020. Most respondents were foreign-born (63.9%), female (78.2%), and monolingual Spanish speakers (63.2%). Results showed that compared to US-born Latinos, foreign-born individuals were older, had lower educational attainment, were most likely to be monolingual Spanish speakers, were low-income, and were more likely to be uninsured. Foreign-born Latinos had lower levels of alcohol consumption and higher consumption of fruits and vegetables. The rate of preventive cancer screenings for breast, cervical and colorectal cancer did not differ by birthplace, although a low fraction (35.3%) of foreign-born Latinas who were up-to-date compared to US-born Latinas (83.3%) with colorectal cancer screening was observed. Time since the last routine check-up for all preventable cancers (cervical p=0.0002, breast p=0.0039, and colorectal p=0.0196) is significantly associated with being up to date with cancer screening. Individuals who had a check-up of two or more years ago are 84% less likely to be up to date with pap smears than those who had a check-up within the year (p=0.0060). Individuals without health insurance are 94% less likely to be up to date with mammograms and colonoscopy/FIT tests (p=0.0016 and p=0.0133, respectively) than those who are insured. There is no significant association between screening and nativity.

**Conclusions:**

Considerable differences in socio-economic and environmental determinants of health and colorectal cancer screening rates were observed between US-born and foreign-born Latinos. The present study represents the foundation for future targeted intervention among immigrant populations at our cancer center’s catchment area.

## Introduction

Cancer is a leading cause of death among Latinos, the largest racial/ethnic minority group in the United States (1). California has the largest Latino population in the country (39%), with most individuals being of Mexican ancestry (2). Relative to non-Latino whites (NLW), Latinos generally have ~25% lower cancer incidence (3).The incidence of common malignancies such as breast, colorectal, lung, and prostate cancers are lower in individuals from this ethnic category, but members of this minority experience higher incidence rates of infection-related cancers like cervical, gastric, and liver compared to NLWs (3). Given the high prevalence of obesity among Latinos, especially among Mexican-Americans, obesity-related cancer incidence rates have increased in recent years (4, 5). Our group and others have also shown that genetic ancestry (acknowledging that Latinos have varying levels of ancestry derived from Europeans, Africans, and Indigenous Americans (6–9) mediates cancer risk and tumor characteristics in this population (10, 11). In addition to infection, obesity, and genetic ancestry risk factors, socio-environmental factors (e.g., socioeconomic status, access to health care, poor diet, physical activity) can also influence the risk profiles in Latinos (3, 12–15).

**Figure 1:**
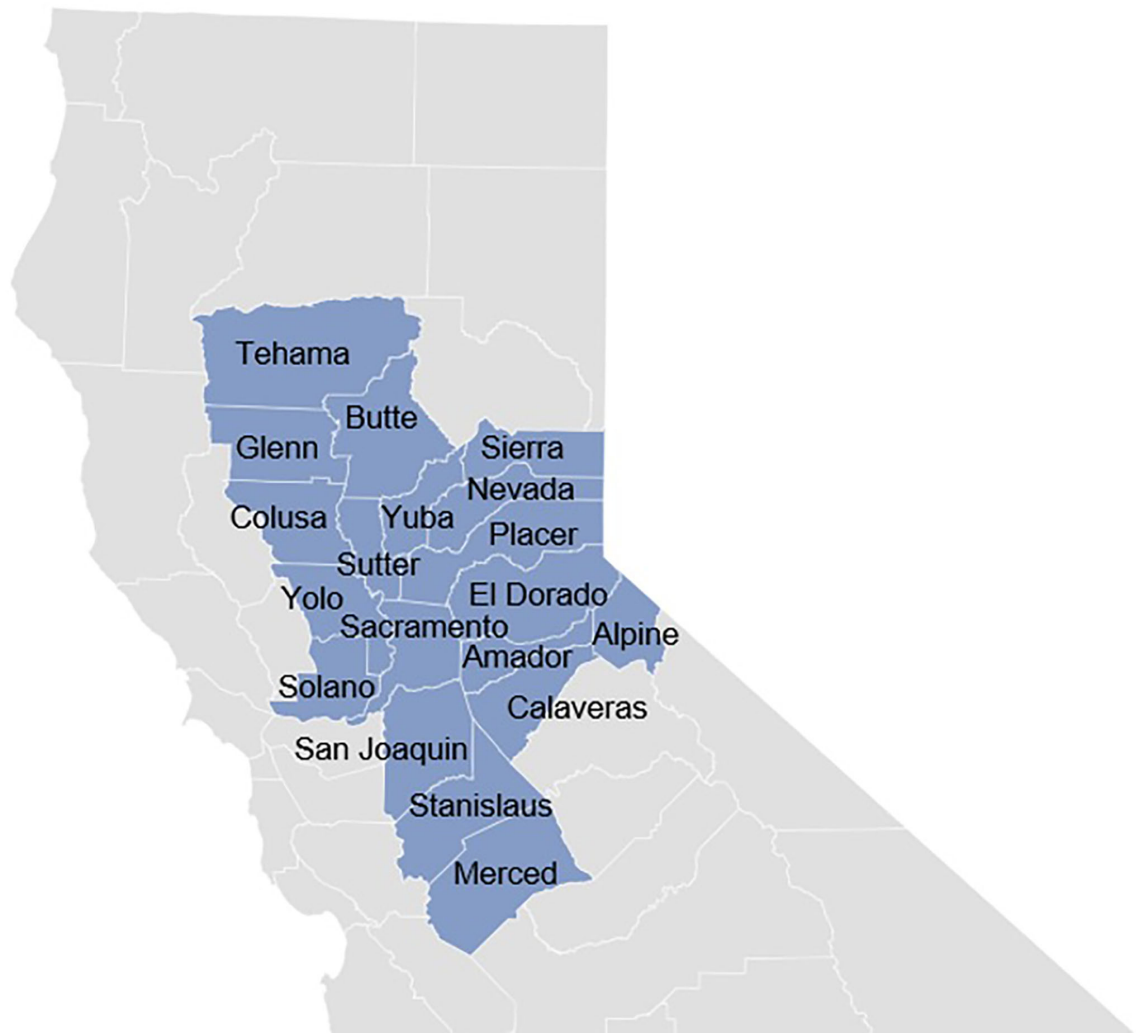
UC Davis Cancer Center 19-County Catchment Area.

The University of California Davis Comprehensive Cancer Center (UCDCCC) has a catchment area with a large Latino population, with counties such as San Joaquin, Merced, Stanislaus, and Colusa, where 50% or more inhabitants have Latino heritage and experience vast socio-economic and environmental disparities (**Figure 1**) (16). A significant fraction of Latinos in these communities are both undocumented and uninsured, representing a major cancer prevention and care challenge. To address health challenges experienced by Latinos in the region, the Latinos United for Cancer Health Advancement (LUCHA) initiative was launched. The goal of LUCHA is to advance health equity in Latino communities through respectful, bi-directional, and community-engaged, translational, clinical, and public health approaches to cancer research. To improve the understanding of local community needs and disentangle cancer health disparities affecting the Latino community, a catchment area health assessment (CAPA) was conducted as an initial LUCHA effort. The CAPA laid the foundation for a strategic plan to better serve the catchment area through health education that emphasizes the importance of public health literacy, early detection, and cancer prevention.

## Materials and Methods

### Measures

A 64-question survey (see details in supplementary material) was developed using validated questions from the National Cancer Institute (NCI) population health assessment supplement (7). Questions in the supplement were reviewed by LUCHA staff, translated to Spanish, edited, and face-validated to ensure that they were culturally appropriate for Latinos residing in California. Data collection took place in the nineteen county UCDCCC catchment area from August 2019 to May 2020. The priority was to capture predictors of cancer screening while also gauging factors influencing the health status of Latinos in the catchment area. The survey included four categories: sociodemographics, lifestyle and behavioral factors, social determinants of health, and cancer screening. Specifically, questions were asked about birthplace, language use, and length of time in the U.S. for foreign-born participants.

Our survey was created in English and was re-written at a 6^th^-grade reading level to ensure it was easy to understand for most adults in the catchment area. The English version was tested in two groups, one consisting of internal members of the UCDCCC Office of Community Outreach and Education and the other of community members with different educational attainment levels. The internal group consisted of eight staff members who all had at least a bachelor’s degree and were of mixed ethnic/racial backgrounds. The external group included all eight members of the UCDCCC’s Community Advisory Board who had a diverse educational backgrounds ranging from high school diplomas to medical degrees and had representation from the following ethnic backgrounds: Asian Americans, Blacks, NLWs, and Latinos.

Once the survey was translated into Spanish by LUCHA native Spanish speakers, the translated survey was completed by ten native Spanish-speaking community members to ensure that it was appropriate for the most common Spanish forms (Mexican and Central American) spoken in the region. The external Spanish-speaking group included people from Mexican, Colombian, and Guatemalan backgrounds with varying levels of education, most of which were high school or GED equivalent and a few with college and graduate-level degrees. The internal group included six bilingual staff members with varying levels of Spanish proficiency, all of whom were college-educated and came from different Latin American countries. During group testing, the time it took to complete the survey was measured since the assumption was that most participants would be answering in person and would not want to spend significant amounts of time filling out the survey. Once feedback was received for both the English and Spanish versions, the team implemented the suggestions and resorted to the second round of group testing internally and with a few external stakeholders. After the second round of testing, the survey was finalized and was sent to the UC Davis IRB for final approval.

### Participant Identification and Eligibility

The participant eligibility criteria included self-identified Latino adults over 18 years living within the UCDCCC catchment area (**Figure 1**). Candidate participants were asked to complete a brief questionnaire to assess eligibility, and verbal consent was obtained for participation. Pre-survey questions included information on birth year, race/ethnicity, and residence zip code.

### Data Collection

The survey was then* *implemented online and in-person (self-administered and coordinator administered using interviewing techniques such as question and answer dialogue) by the LUCHA bilingual-bicultural team. Data was initially collected in community settings, including outdoor health and wellness events, churches, and partnerships with community clinics, agencies, and family centers. This initial effort resulted in over 200 completed surveys at 17 community events. However, in March 2020, due to the COVID-19 pandemic lockdown, the collection effort was redirected to solely online collection, with the dissemination of survey links occurring through community partners, listservs, online classes, and social media. Surveys were available in both English and Spanish. For surveys collected in person, participants were approached by LUCHA staff to participate. Consent was obtained either verbally and/or in writing. Participants were also given the option to complete the survey at home by taking a flyer with a link to the survey. All study data collected was managed using the Qualtrics Research Suite, a web-based survey tool. Fifty-one surveys were collected online, and 204 were done in person and entered by staff.

### Statistical Analysis

We used descriptive statistics to describe socio-demographic variables, health characteristics and behaviors, and cancer screening. As seen in the tables, numbers do not reflect the total number of participants, given that some questions were left unanswered. Denominators reflect those that answered the questions. Analyses focused on comparisons of foreign-born versus US-born Latinos. Chi-square and Fisher’s exact tests were used to assess group differences with two-sided tests and a significance level of 0.05. Cancer screening proportions were calculated using recommended age ranges by national guidelines. Multivariable logistic regression models were used to identify variables associated with cancer screening. We used Akaike Information Criterion (AIC) to select optimal variables. Age was kept as a confounding variable, and nativity was kept as a variable of interest. We calculated unadjusted and adjusted odds ratios (ORs) and 95% confidence intervals (CI) for numerous binary outcomes. Model performance for cancer screenings was assessed using the area under the curve (AUC) analysis under R library pROC() function with bootstrapping technique. Average AUC and 95% confidence intervals are calculated with bootstrap replicates to estimate the second significant digit of the confidence interval.SAS v9.4 and R Studio v4.0.0 were used to conduct statistical analyses.

## Results

### Socio-Demographics

255 participants completed the surveys and were included in the analysis. A high fraction of participants were foreign-born Latinos (63.9%), female (78.2%), and monolingual Spanish speakers (63.2%). The average age of foreign-born participants was higher than US-born (44.7 versus 36.1, p<0.0001, **Table 1**). In general, foreign-born respondents had lower educational attainment, a higher fraction of monolingual Spanish speakers. They reported a lower opinion of their English-speaking ability compared to US-born respondents (21.9% of foreign-born reported speaking English very well vs. 84.6% among US-born participants: p<0.0001, see **Table 1**). Moreover, while over 50% of individuals in both groups reported being employed, foreign-born respondents had a larger proportion of homemakers (24.7% vs. 1.2%, p<0.0001). In contrast, US-born respondents included more students (18.3% vs. 6.3%, p<0.0001, **Table 1**). A larger fraction of foreign-born individuals had an annual income of less than $35,000/year (52.1% vs. 35.9%, p=0.0126).

**Table 1:** Demographics of participants stratified by nativity. *(N=246)*.

Variable	Overall *(N=255)*	U.S. Born *(N=83)*	Foreign-Born *(N=163)*	p-value
**Age Group**				
18-30y	67 (27.5%)	36 (47.4%)	27 (17.0%)	<0.0001*
31-40y	60 (24.6%)	17 (22.4%)	42 (26.4%)	
41-50y	53 (21.7%)	12 (15.8%)	40 (25.2%)	
51-65y	52 (21.3%)	7 (9.2%)	42 (26.4%)	
66y+	12 (4.9%)	4 (5.3%)	8 (5.0%)	
**Mean Age (sd)**	41.9 (14.1)	36.1 (15.2)	44.7 (12.7)	<0.0001**
**Sex**				
Male	55 (21.8%)	18 (21.7%)	37 (23.1%)	0.7994
Female	197 (78.2%)	65 (78.3%)	123 (76.9%)	
**Language Spoken at Home**				
English	41 (16.4%)	29 (35.4%)	10 (6.3%)	**<0.0001**
Spanish	158 (63.2%)	20 (24.4%)	134 (84.3%)	
Both	51 (20.4%)	33 (40.2%)	15 (9.4%)	
**English Speaking Ability**				
Very Well	72 (38.7%)	44 (84.6%)	28 (21.9%)	<0.0001*
Well	39 (21%)	7 (13.5%)	30 (23.4%)	
Not Well	64 (34.4%)	1 (1.9%)	61 (47.7%)	
Not At All	11 (5.9%)	0 (0.0%)	9 (7.0%)	
**Education**				
<High school	61 (25.0%)	3 (3.7%)	52 (34.0%)	<0.0001*
High school graduate	57 (23.4%)	16 (19.5%)	39 (25.5%)	
Some college/Vocational	65 (26.6%)	30 (36.6%)	34 (22.2%)	
College grad or higher	61 (25.0%)	33 (40.2%)	28 (18.3%)	
**Occupational Status**				
Employed	140 (57.1%)	51 (62.2%)	87 (55.1%)	<0.0001*
Student	25 (10.20%)	15 (18.3%)	10 (6.3%)	
Homemaker	40 (16.3%)	1 (1.2%)	39 (24.7%)	
Unemployed/Disabled/Retired	40 (16.3%)	15 (18.3)	22 (13.9%)	
**Family Annual Income**				
<$35k	107 (47.1%)	28 (35.9%)	74 (52.1%)	**0.0126**
$35k-$74.9k	78 (34.4%)	28 (35.9%)	49 (34.5%)	
$75k+	42 (18.5%)	22 (28.2%)	19 (13.4%)	

Two-sided p-values form Chi-square test are used, unless a cell had less than 10, Fisher’s exact test was used. *denotes Fischer’s exact test. **denotes Independent T-Test for continuous variables. Bolded p-value indicates significance level of p<0.05.

### Health Characteristics and Behaviors

Most participants had health insurance (72.4%). However, the fraction of uninsured was significantly higher among foreign-born than US-born (38.6% vs. 5.2%, p<0.0001, **Table 2**) participants. Only 12.7% of participants reported not having a location to obtain regular healthcare services, and 84.5% of all participants had been seen for a routine check-up in the last two years (**Table 2**). For the location of health care services, foreign-born individuals more commonly went to community clinics/healthcare centers (52.3% vs. 37.2%), emergency rooms (3.4% vs. 2.6%), or some other place (2.6% vs. 0.0%) than their US-born counterparts, who instead reported going to the doctor’s office or used HMO most often (52.6% vs. 20.5%, p<0.0001, **Table 2**). Significant differences were not seen between the two groups regarding delayed medical treatment within the last year, opinion of health condition, or confidence in getting medical information.

**Table 2:** Health characteristics of participants stratified by nativity. (*N = 246)*.

Variable	Overall *(N=255)*	U.S. Born (*N=83)*	Foreign-Born (*N=163)*	p-value
**Health Insurance**				
Private	124 (54.4%)	52 (67.5%)	70 (48.3%)	<0.0001*
Public	31 (13.6%)	14 (18.2%)	16 (11.0%)	
Some other Source	10 (4.4%)	7 (9.1%)	3 (2.07%)	
None	63 (27.6%)	4 (5.2%)	56 (38.6%)	
**Location to get health care services**				
Clinic or health center	122 (51.7%)	29 (37.2%)	88 (52.3%)	<0.0001*
Doctor’s office or HMO	72 (30.5%)	41 (52.6%)	31 (20.5%)	
Hospital emergency room	8 (3.4%)	2 (2.6%)	6 (4.0%)	
Some other place	4 (1.7%)	0 (0.0%)	4 (2.6%)	
There is no place	30 (12.7%)	6 (7.7%)	22 (14.6%)	
**Time Since Last Routine Check-up**				
<1y	169 (70.7%)	62 (78.5%)	103 (66.9%)	0.1298*
1-2y	33 (13.8%)	11 (13.9%)	21 (13.6%)	** **
2-5y	13 (5.4%)	3 (3.8%)	10 (6.5%)	
5y+	16 (6.7%)	3 (3.8%)	12 (7.8%)	
Never	8 (3.3%)	0 (0.0%)	8 (5.2%)	
**Traveling to Another Country for Medical Care**				
Yes	34 (13.7%)	9 (11.1%)	25 (15.4%)	0.4352*
No	214 (86.3%)	72 (88.9%)	137 (84.6%)	
**Delayed Medical Care in the Last 12 Months**				
Yes	70 (30.2%)	21 (26.6%)	47 (31.8%)	0.4175
No	162 (69.8%)	58 (73.4%)	101 (68.2%)	
**Opinion of Health Condition**				
Excellent	19 (7.9%)	8 (9.8%)	10 (6.6%)	0.7256*
Very Good	43 (17.9%)	17 (20.7%)	26 (17.2%)	
Good	108 (22.1%)	34 (41.5%)	70 (46.4%)	
Fair	53 (22.1%)	19 (23.2%)	33 (21.9%)	
Poor	17 (7.1%)	4 (4.9%)	12 (7.9%)	
**Confidence in Getting Medical Information**				
Completely Confident	60 (25.0%)	30 (37.0%)	30 (19.7%)	0.0744*
Very Confident	68 (28.3%)	20 (24.7%)	46 (30.3%)	
Somewhat confident	71 (29.6%)	21 (25.9%)	48 (31.6%)	
A little confident	30 (12.5%)	7 (8.6%)	22 (14.5%)	
Not Confident at all	11 (4.6%)	3 (3.7%)	6 (3.9%)	
**Hep B Vaccine**				
At least 3 doses	80 (57.6%)	36 (67.9%)	43 (53.1%)	0.0098*
Less than 3 doses	13 (9.4%)	7 (13.2%)	4 (4.9%)	
No doses	46 (33.1%)	10 (18.9%)	34 (42.0%)	
**Current Smoker**				
Yes	18 (7.6%)	6 (7.3%)	12 (7.8%)	1.0000*
No	218 (92.4%)	76 (92.7%)	141 (92.2%)	
**Alcohol Consumption in the Last 30 Days**				
Yes	65 (28.8%)	32 (42.1%)	33 (23.1%)	**0.0033**
No	161 (71.2%)	44 (57.9%)	110 (76.9%)	
**Atleast One Serving of Fruit/Day**				
Yes	148 (66.7%)	41 (53.2%)	104 (74.3%)	**0.0016**
No	74 (33.3%)	36 (46.8%)	36 (25.7%)	
**Atleast One Serving of Vegetables/Day**				
Yes	133 (60.7%)	44 (57.1%)	87 (63.5%)	0.3594
No	86 (39.3%)	33 (42.9%)	50 (36.5%)	
**Exercise per Week (minimum 20min)**				
0-3 days	98 (46.0%)	31 (43.1%)	63 (47.0%)	0.5864
4-7 days	115 (54.0%)	41 (56.9%)	71 (53.0%)	
**BMI**				
Normal	51 (23.9%)	24 (30.8%)	27 (20.8%)	0.1251
Overweight	63 (29.6%)	17 (21.8%)	43 (33.1%)	
Obese	99 (46.5%)	37 (47.4%)	60 (46.2%)	
**Malignancy Identified by a Health Professional**				
Yes	26 (10.6%)	7 (8.5%)	18 (11.4%)	0.6566*
No	220 (89.4%)	75 (91.5%)	140 (88.6%)	
**Family History of Cancer**				
Yes	118 (51.1%)	35 (45.5%)	80 (53.7%)	0.2404
No	113 (48.9%)	42 (54.5%)	69 (46.3%)	

Two-sided p-values from Chi-square test are reported, unless a cell had less than 10, then Fisher’s exact test was used. *denotes Fischer’s exact test. Bolded p-value indicates significance level of p<0.05.

Analysis of health behaviors showed that nearly 60% of the participants completed the Hepatitis B vaccine series (**Table 2**), with a lower fraction among foreign-born participants (67.9% for US-born vs. 53.1% for foreign-born; p=0.0098, **Table 2**). In general, foreign-born Latinos had healthier lifestyles and diets compared to US-born, with reported lower alcohol consumption (23.1% vs. 42.1%, p=0.0033), and more consumption of fruits (74.3% vs. 53.2%, p=0.0016) and vegetables (63.5% vs. 57.1%, p=0.3594, **Table 2**). No differences were observed in exercise, BMI, and history of cancer (personal and familial).

### Cancer Screening Rates

**Figure 2:**
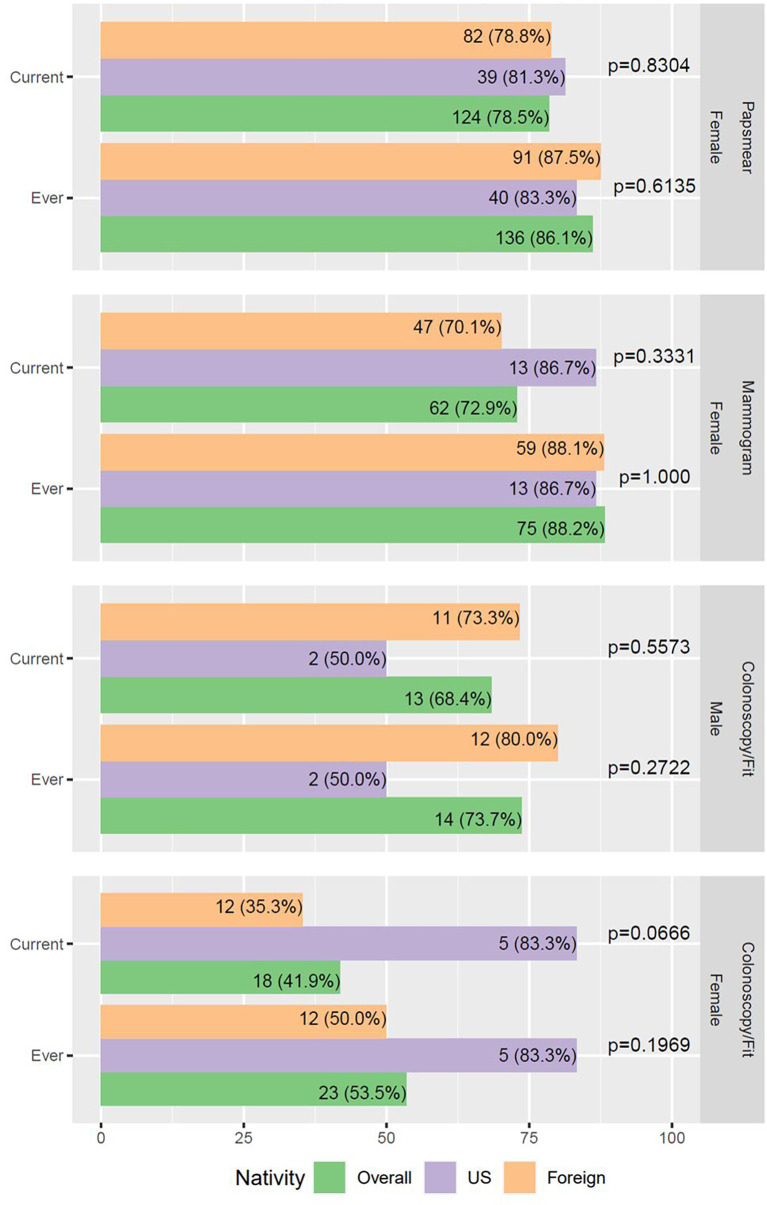
Cancer screening rates in survey participants. Two-sided p-values from Chi-square test or Fisher’s exact test are reported. Age inclusion: 21-65yo females for papsmear (N = 158); 40-75yo females for mammogram (N = 85); 50-75yo for colonoscopy/FIT (N = 43).

As fewer respondents were old enough to assess their adherence to cancer screenings, our study had limited power to detect differences in screening rates between foreign-born and US-born Latinos. We, however noted that most female participants were up-to-date with pap smears and mammograms (78.5% and 72.9%, respectively, **Figure 2**). The foreign-born group had a lower fraction of women up-to-date with their breast cancer screening (70.1% vs. 86.7%), although this difference was not significant. A low fraction of female participants had colorectal cancer screening (53.5%) or were up to date with screening (41.9%). Furthermore, a lower fraction of foreign-born Latinas was up to date with such screening (35.3% vs. 83.3%, p=0.0666, **Figure 2**).

### Multivariable Predictors of Cancer Screening Rates

After adjusting for age, education, health insurance, and place of birth, it was found that the longer it had been since a person had their last routine check-up, the lower their odds of being up-to-date with cervical and breast cancer screenings. For cervical cancer screening, individuals are less likely to be up to date by 58% (p=0.1615) if they had their last check-up after one year and by 84% (p=0.0060) if they had their last check-up after two years compared to individuals who had their check-up within the year (**Table 3**).

**Table 3:** Unadjusted and adjusted odds ratios (OR) from logistic regression for factors associated with cervical, breast and colorectal cancer screening.

	Cervical Cancer			Breast Cancer			Colorectal Cancer		
Variable	Unadjusted	Adjusted	p-value	Unadjusted	Adjusted	p-value	Unadjusted	Adjusted	p-value
**Age (continuous)**	1.04 (1.00-1.08)	1.09 (1.03-1.15)	**0.0038**	1.12 (1.02-1.22)	1.10 (0.97-1.25)	0.1199	1.27 (1.1-1.45)	1.24 (1.05-1.46)	**0.0101**
**Education**									
Some college or more	1.00 (ref)	1.00 (ref)		1.00 (ref)	1.00 (ref)		1.00 (ref)	1.00 (ref)	
High school or less	0.37 (0.17-0.83)	0.23 (0.08-0.7)	**0.0092**	0.55 (0.18-1.67)	1.05 (0.22-4.94)	0.9501	0.32 (0.1-0.97)	0.37 (0.08-1.70)	0.1997
**Time Since Last Routine Check-up**									
≤ 1 year	1.00 (ref)	1.00 (ref)		1.00 (ref)	1.00 (ref)		1.00 (ref)	1.00 (ref)	
1-2 years	0.30 (0.11-0.83)	0.42 (0.12-1.41)	0.1615	0.24 (0.06-1.04)	0.64 (0.09-4.64)	0.6590	0.66 (0.04-11.12)	NE	
2 or more years	0.15 (0.05-0.41)	0.16 (0.04-0.59)	**0.0060**	0.14 (0.04-0.51)	0.34 (0.05-2.38)	0.2753	0.09 (0.01-0.82)	NE	
**Place of birth**									
U.S. born	1.00 (ref)	1.00 (ref)		1.00 (ref)	1.00 (ref)		1.00 (ref)	1.00 (ref)	
Foreign born	0.86 (0.36-2.04)	0.84 (0.24-2.92)	0.7843	0.43 (0.09-2.08)	3.73 (0.4-34.86)	0.2487	0.41 (0.09-1.78)	1.30 (0.21-8.07)	0.7794
**Health Insurance**									
Yes	1.00 (ref)	1.00 (ref)		1.00 (ref)	1.00 (ref)		1.00 (ref)	1.00 (ref)	
No	0.59 (0.26-1.38)	0.82 (0.26-2.62)	0.7425	0.11 (0.04-0.37)	0.06 (0.01-0.34)	**0.0016**	0.03 (0.00-0.28)	0.06 (0.01-0.55)	**0.0133**

NE: Not estimated to avoid model overfitting due to small sample size. P-values for the adjusted model are reported, unadjusted p-values not shown. Bolded p-value indicates significance level of p<0.05.

When adjusting for education, check-up (not included in colorectal model), health insurance, and place of birth, each one-year increase in age resulted in individuals being 1.09 (1.03-1.15, p=0.0038), 1.10 (0.97-1.25, p=0.1199), and 1.24 (1.05-1.46, p=0.0101) times more likely to be up to datefor cervical, breast, and colon cancer screening (**Table 3**). No statistical association was found between the place of birth and cancer screening rates.

Those who do not have health insurance are less likely to be up to date with cancer screenings than individuals who have health insurance. For breast and colorectal screenings, individuals with no health insurance were 94% less likely to be up to date (p=0.0016 and p=0.0133, **Table 3**).

**Figure 3:**
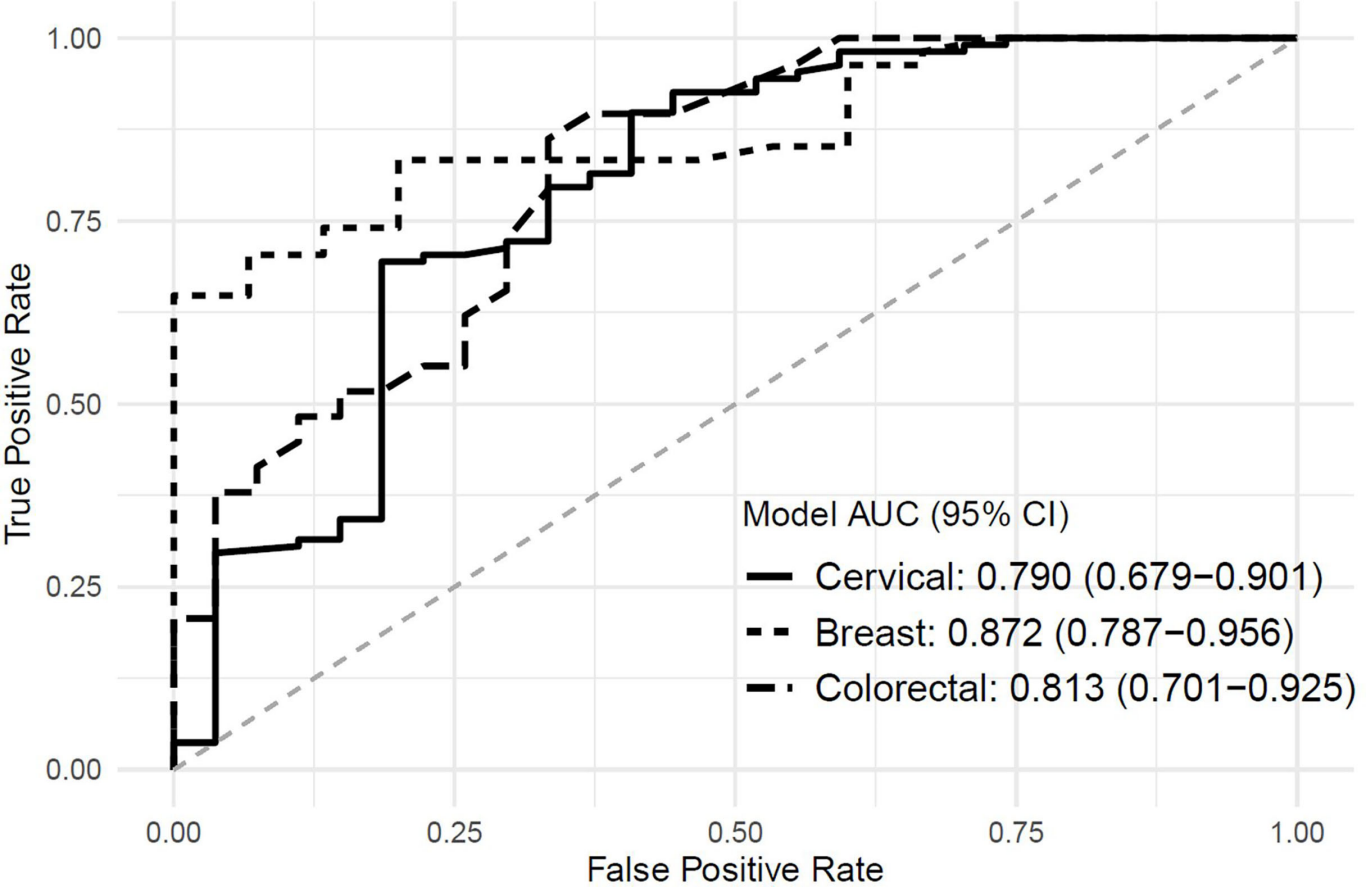
Area Under the curve for multiple logistic regression screening models.

Our breast screening model has the best performance at area under the curve (AUC) = 0.872 (0.787-0.956) followed by colorectal screening at AUC = 0.813 (0.701-0.925) and cervical screening at AUC = 0.790 (0.679-0.901). Using a person’s age, education, nativity, health insurance, and time since the last routine check-up (for cervical and breast, not in the colorectal model) yields acceptable model accuracies (**Figure 3**).

## Discussion

To our knowledge, LUCHA is one of the few initiatives at NCI-designated cancer centers that solely focus on advancing Latino cancer health equity. The current study is also the first Latino-population-focused CAPA.

This cancer health needs assessment showed that while individuals who identify as Latino are grouped into one racial/ethnic category, there are substantial differences between foreign-born and US-born Latinos. In California, the state with the largest Latino population, about 13.2% are foreign-born, of which 59% are undocumented immigrants from Latin America, making this population particularly harder to reach by the cancer center’s community outreach and engagement programs (17). As seen in **Table 1**, the survey showed that foreign-born Latinos were more likely to have lower levels of education, lower levels of English fluency, make less than $35,000/year, and lack healthcare insurance. This reiterates the notion that the relationship between health and the Latino population is very complex (13). For example, a 2017 poll reported that 20% of Latinos experienced discrimination in a healthcare setting, and 17% relayed that they had avoided seeking medical care for themselves or family members due to concerns of being discriminated against or being treated poorly (18, 19). The fear of discrimination, alongside the anxiety of possible deportation among Latino families with mixed statuses, discourages families from seeking health services, leading to later cancer diagnosis, treatment, and outcome (15, 20).

Both social-ecological factors and ethnic heterogeneity play a role in cancer risk and mortality. Despite these differences, there was no association between nativity and cancer screenings among participants of the CAPA survey. Seventy percent or more of our survey respondents were current with their screenings for both breast and cervical cancer (**Figure 2**). However, for colorectal cancer screening, the fraction of up-to-date respondents was significantly smaller, even with a small sample size of screening eligible respondents. **Supplementary Table 1** demonstrates that while nativity was not associated with screening, sociodemographic factors like college education were associated with increased screening adherence. This is not surprising given that college education is linked to an increase in social status and income, which is also linked to better health insurance and, thus, the ability to seek care.

Limitations of this study include the sample size (N=255), the polarized political climate (e.g., national and state election campaigns), a heightened media focus on race issues during the survey period, and the COVID-19 pandemic (15). While the sample size for the current study is relatively small, interesting differences were uncovered that will be explored in future work. Additionally, while the survey did not ask for Protected Health Information (PHI), given the political climate, many individuals might have been hesitant to answer the survey due to fear or anxiety. The COVID-19 pandemic also altered the ability of surveyors to fan out into the community; subsequently, there was a rise in the completion of online surveys when the methodology was changed to a classroom setting (administered to UC Davis undergraduate students), which may explain a younger US-born sample and reflects some sample bias. With a younger group of survey participants, some of them may not have been eligible for cancer screenings which impacts the results. We also did not follow the participants for a period of time and were unable to do a trend analysis.

The rate at which the Latino population is growing and their increasing numbers of new cancer cases make them a critical public health priority. In California, the public health concern lies in the economic burden of cancer care and the unique challenges Latinos face in different geographical areas. The findings reported here suggest a need for more efforts to create health equity and add to current literature that supports the notion that there are differences in healthcare utilization and access among Latinos. At UC Davis, this means bringing health screenings to the community in the form of mobile clinics, vaccine clinics, and hosting health education workshops while patients wait for appointments. We understand that it is unrealistic to expect individuals with limited educational attainment and language fluency to navigate such a complex health system. Moreover, despite the advances in cancer care, Latinos face many different structural and social challenges that influence their cancer prognosis. It is important to understand the gaps in cancer awareness and care access to eliminate cancer health disparities.

## Data Availability Statement

The raw data supporting the conclusions of this article will be made available by the authors, without undue reservation.

## Ethics Statement

The studies involving human participants were reviewed and approved by UC Davis IRB. The patients/participants provided their written informed consent to participate in this study.

## Author Contributions

JQ, FP, AP, and AV contributed equally. LC-C led the conception and design of the study. FP, AP, JQ, SR, and AM aided in study recruitment. JQ and AV organized the database. JQ, AV, LA, and MN performed the statistical analysis. JQ wrote the first draft of the manuscript. JD, MC, LF, and PL gave input throughout the process. LC-C served as head PI. All authors contributed to manuscript revision, read, and approved the submitted version.

## Funding

This study was funded with support from the UC Davis Cancer Center Support Grant from the National Cancer Institute (NCI, P30CA093373) and the UC Davis Office of the Provost (Latinos United for Cancer Health Advancement, LUCHA, Initiative). JQ is supported by a diversity supplement from the National Cancer Institute (U54CA233306-1S) of the National Institutes of Health (NIH). JD receives support from NCI (P30CA093373), Bristol-Myers Squibb Foundation (A182016001), NIA (R61AG068948), and USDHHS (1CPMP19176). LF and LC-C received support from the California Initiative to Advance Precision Medicine (contract OPR18111). LC-C cancer health disparities and community engagement efforts receive support from NCI (P30CA093373, U54CA233306, and R01CA223978), National Center for Advancing Translational Science (UL1TR001860), the Heart, BrEast, and BrAin HeaLth Equity Research (HEAL-HER) Program and the Auburn Community Cancer Endowed Chair in Basic Science.

## Author Disclaimer

The opinions expressed in this article are the author’s own and do not reflect the view of the National Institutes of Health, the Department of Health and Human Services, or the United States government.

## Conflict of Interest

The authors declare that the research was conducted in the absence of any commercial or financial relationships that could be construed as a potential conflict of interest.

## Publisher’s Note

All claims expressed in this article are solely those of the authors and do not necessarily represent those of their affiliated organizations, or those of the publisher, the editors and the reviewers. Any product that may be evaluated in this article, or claim that may be made by its manufacturer, is not guaranteed or endorsed by the publisher.
