# Supplementary material for: A Cancer Health Needs Assessment Reveals Important Differences Between US-Born and Foreign-Born Latinos in California
Source: Front Oncol. 2022 Jul 7;12:883200. doi: 10.3389/fonc.2022.883200 (PMC9300947; doi:10.3389/fonc.2022.883200)
Supplement: Supplementary file 1 [file Table_1.docx]

*Data Cleaning*

Data cleaning was performed at the end of the collection period. A total of 307 survey entries were captured, however, only 255 surveys were analyzed. Discarded surveys included attempted survey entries that were captured by Qualtrics or in-person but did not meet eligibility such as ethnicity (n=2) or lived outside the area of interest (n=18). This occurred due to two reasons. For online entries, Qualtrics retained any survey attempt, even when participants did not meet the eligibility and were unable to continue. For in person-surveys, if a coordinator did not assist a participant with the survey, eligibility criteria was sometimes overlooked by the participant and surveys would be returned. Surveys that were less than 33% complete (completed only up to demographics, n=32) were also removed.

Participants were grouped into two categories: U.S. born and foreign-born (place of birth outside of U.S. territory). Health insurance was also subset to two categories, public (Medi-Cal) and private (Medicare, a plan purchased through an employer or union, and a plan that you or another family member buys on their own), after an individual answered whether they had insurance or not. Similarly, if individuals answered “yes” to having a location to seek health services, we then focused on where they went for services. To calculate the current smoking rate, we collapsed two questions together. If an individual answered “yes” to question #35 and either “every day” or some days” to question #36 or said “no” to question #35 and either “every day” or some days” to question #36, then they were considered a current smoker. For alcohol consumption, if they reported drinking any amount of alcohol in the last 30 days, it was considered “yes.” Fruit and vegetable consumption were averaged to daily amounts from participant answers (they were given the option to write their amount by day, week, and monthly).

*Table*

| **Supplementary Table 1.** Risk factors of cervical, breast, and colorectal cancer screening. | | | | | | | | | |
| --- | --- | --- | --- | --- | --- | --- | --- | --- | --- |
|  | ***Cervical Cancer*** | | | ***Breast Cancer*** | | | **Colorectal Cancer** | | |
| **Variables** | **Yes, N = 124 (78.5%)** | **No, N = 34 (21.5%)** | **p-value** | **Yes, N = 62 (75.6%)** | **No, N=20 (24.4%)** | **p-value** | **Yes N = 31 (51.7%)** | **No, N=29 (48.3%)** | **p-value** |
| **Place of Birth** |  |  | 0.8304 |  |  | 0.5021 |  |  | 0.3044 |
| U.S. born | 39(32.2%) | 9(29.0%) |  | 13(21.7%) | 2(10.5%) |  | 7(23.3%) | 3(11.1%) |  |
| Foreign born | 82(67.8%) | 22(71.0%) |  | 47(78.3%) | 17(89.5%) |  | 23(76.7%) | 24(88.9%) |  |
| **Education** |  |  | **0.0037** |  |  | 0.3584 |  |  | 0.1188 |
| <High school | 27 (22.7%) | 6(18.2%) |  | 18(30.5%) | 9(50.0%) |  | 7(23.3%) | 14(48.3%) |  |
| High school graduate | 20(16.8%) | 15(45.5%) |  | 13(20.0%) | 3(16.7%) |  | 8(26.7%) | 8(27.6%) |  |
| Some college or vocational school | 36(30.3%) | 9(27.3%) |  | 16(27.1%) | 5(27.8%) |  | 10(30.3%) | 6(20.7%) |  |
| College graduate or higher | 36(30.3%) | 3(9.1%) |  | 12(20.3%) | 1(5.5%) |  | 5(16.7%) | 1(3.4%) |  |
| **Language Spoken at Home** |  |  | 0.8395 |  |  | 0.7805 |  |  | 0.1479 |
| English or both | 45(37.2%) | 12(35.3%) |  | 19(31.1%) | 5(26.3%) |  | 11(35.5%) | 5(17.2%) |  |
| Spanish | 76(62.8%) | 22(64.7%) |  | 42(68.9%) | 14(73.9%) |  | 20(64.5%) | 24(82.8%) |  |
| **Annual Income** |  |  | 0.6566 |  |  | 0.2352 |  |  | 0.0718 |
| <$50k | 74(64.9%) | 20(71.4%) |  | 35(63.6%) | 13(81.3%) |  | 18(62.1%) | 23(85.2%) |  |
| $50k+ | 40(35.1%) | 8(28.6%) |  | 20(36.4%) | 3(18.8%) |  | 11(24.1%) | 4(14.8%) |  |
| **Occupational Status** |  |  | 0.2385 |  |  | 0.7910 |  |  | 0.9222 |
| Employed | 51(42.1%) | 18(54.5%) |  | 25(41.0%) | 9(47.4%) |  | 17(54.8%) | 15(53.6%) |  |
| Not employed | 70(57.9%) | 15(45.5%) |  | 36(59.0%) | 10(52.6%) |  | 14(45.2%) | 13(46.4%) |  |
| **Health Insurance Type** |  |  | 0.0795 |  |  | 0.2589 |  |  | 0.1843 |
| Private | 62(72.1%) | 15(75.0%) |  | 41(85.4%) | 4(66.7%) |  | 27(90.0%) | 10(71.4%) |  |
| Public/Other | 24(27.9%) | 5(25.0%) |  | 7(14.6%) | 2(33.3%) |  | 3(10.0%) | 4(28.6%) |  |
| **Time Since Last Routine Check-up** | |  | **0.0002** |  |  | **0.0039** |  |  | **0.0196** |
| ≤ 1 year | 93(77.5%) | 14(42.4%) |  | 50(80.6%) | 8(50.0%) |  | 29(93.5%) | 19(70.4%) |  |
| 1-2 years | 16(13.3%) | 8(24.2%) |  | 6(9.7%) | 4(21.1%) |  | 1(3.2%) | 1(3.7%) |  |
| 2 or more years | 11(9.2%) | 11(33.3%) |  | 6(9.7%) | 7(36.8%) |  | 1(3.2%) | 7(25.9%) |  |
| **Location of Health Services** |  |  | 0.0795 |  |  | 0.0620 |  |  | 0.2335 |
| Cinic or health center | 64(52.9%) | 15(48.4%) |  | 25(43.5%) | 14(73.7%) |  | 10(34.5%) | 14(51.9%) |  |
| Doctor's office or HMO | 42(34.7%) | 7(22.6%) |  | 24(38.7%) | 3(15.8%) |  | 13(44.8%) | 6(22.2%) |  |
| Other/None | 15(12.4%) | 9(29.0%) |  | 11(17.7%) | 2(10.5%) |  | 6(20.7%) | 7(25.9%) |  |
| **Health insurance** |  |  | 0.2233 |  |  | **0.0002** |  |  | **<0.0001** |
| Yes | 90(76.3%) | 21(65.6%) |  | 48(81.4%) | 6(33.3%) |  | 30(96.8%) | 14(50.0%) |  |
| No | 28(23.7%) | 11(34.4%) |  | 11(18.6%) | 12(66.7%) |  | 1(3.2%) | 14(50.0%) |  |
| Note: Two-sided p-value from Chi-square test is reported unless a cell had less 10, then Fisher's exact test was used. | | | | | | | | | |

*Survey*

| **Eligibility Pre-screening Questions**  **Please write in, mark or fill in the circle next to your answer for each question.** | | | | | | | |  |
| --- | --- | --- | --- | --- | --- | --- | --- | --- |
| 1. **Are you at least 18 years of age?** | | | |  | | **5. What county do you live in?** | |  |
| - - Yes   - No | |  | |  | | - Alpine - Amador - Butte - Calaveras - Colusa - El Dorado - Glenn - Merced - Nevada - Placer | - Sacramento - San Joaquin - Sierra - Solano - Stanislaus - Sutter - Tehama - Yolo - Yuba - Other |  |
| 1. **Are you Hispanic or Latino?** | | | |  | |  |  |  |
| - Yes - No | |  | |  | |  |  |  |
| 1. **What is your race/ethnicity? One or more categories may be selected. Mark all that apply.** | | | |  | |  |  |  |
| - South American | |  | |  | | **6. What is your zip code?** | | |
| - Central American - Mexican - Dominican Republic - Puerto Rican - Cuban - Other: ________________ - Don't know - Refuse to answer | |  | |  | | __________________________________  **7. What year were you born?**  __________________________________ | | |

Survey Questionnaire

| **8. Are you male or female?** |
| --- |

- - Male
  - Female
  - Don’t know
  - Refuse to answer

| **9. What is the highest grade or level of schooling you completed?** |
| --- |

- - Less than 8 years
  - 8 through 11 years
  - 12 years or completed high school
  - Post high school training other than college (vocational or technical)
  - Some college
  - College graduate
  - Postgraduate
  - Don't know
  - Refuse to answer

| **10. What language do you speak at home?** |
| --- |

- - English (skip to #12)
  - Spanish
  - Other (specify: _________________________)
  - Don't know
  - Refuse to answer

| **11. Since you speak a language other than English at home, we are interested in your own opinion of how well you speak English. Would you say you speak English?** |
| --- |

- - Very well
  - Well
  - Not well
  - Not at all
  - Don’t know
  - Refuse to answer

| **12. Thinking about members of your family living in this household, what is your combined annual income, meaning the total pre-tax income from all sources earned in the past year?** | | |
| --- | --- | --- |
| - Less than $10,000 - 10,000 to under $15,000 - $15,000 to under $20,000 - $20,000 to under $35,000 - $35,000 to under $50,000 - $50,000 to under $75,000 | | - - $75,000 to under $100,000   - $100,000 to under $200,000   - $200,000 or more   - Don't know/not sure   - Refuse to answer |

| **13. Do you have any kind of health care coverage, including health insurance, prepaid plans such as HMOs, government plans such as Medicare, or Indian Health Service?** | | | | | | | | | | | | | | | | | | | | |
| --- | --- | --- | --- | --- | --- | --- | --- | --- | --- | --- | --- | --- | --- | --- | --- | --- | --- | --- | --- | --- |
| - Yes | | | | | - No | | | | - Don't know/ not sure | | | - Refuse to answer | | | | | |  |  |  |
|  | | | | | | | | | | | | | | | | | | | | |
| **14. If you answered yes, what type of health care coverage do you have?** | | | | | | | | | | | | | | | | | | | | |
| - - A plan purchased through an employer or union (includes plans purchased through another person's employer) | | | | | | | | | | | | | | | | | | | | |
| - - A plan that you or another family member buys on your own | | | | | | | | | | | | | | | | | | | | |
| - - Medicare | | | | | | | | | | | | | | | | | | | | |
| - - Medicaid or other state program | | | | | | | | | | | | | | | | | | | | |
| - - Tricare (formerly CHAMPUS), VA, or Military | | | | | | | | | | | | | | | | | | | | |
| - - Alaska Native, Indian Health Service, Tribal Health Services | | | | | | | | | | | | | | | | | | | | |
| - - Some other source | | | | | | | | | | | | | | | | | | | | |
| - - None (no coverage) | | | | | | | | | | | | | | | | | | | | |
| - - Don't know/not sure | | | | | | | | | | | | | | | | | | | | |
| - - Refuse to answer | | | | | | | | | | | | | | | | | | | | |
| **15. What is your current occupational status? Mark only one.** | | | | | | | | | | | | | | | | | | | | |
| - - Employed, full time | | | | | | | | | | | | | - - Self Employed | | | | | | | |
| - - Employed, part time | | | | | | | | | | | | | - - Retired | | | | | | | |
| - - Unemployed | | | | | | | | | | | | | - - Disabled | | | | | | | |
| - - Homemaker | | | | | | | | | | | | | - - Other________ | | | | | | | |
| - - Student | | | | | | | | | | | | | - - Don't know | | | | | | | |
|  | | | | | | | | | | | | | - - Refuse to answer | | | | | | | |
| **16. Have you ever looked for information about health or medical topics from any source?** | | | | | | | | | | | | | | | | | | | | |
| - Yes | | | | | | - No | | | | | | - Don't know | | | - Refuse to answer | | | | |  |
| **17. If yes, the most recent time you looked for information about health or medical topics, where did you go first?** | | | | | | | | | | | | | | | | | | | | |
| - Books   - Brochures, pamphlets, etc.   - Cancer organization   - Family   - Friend/coworker   - Doctor or health care provider   - Internet   - Library | | | | | | | | - Magazines - Newspapers - Telephone information number - Complementary, alternative, or unconventional practitioner - Social media site, such as Facebook, patients like me, caring bridge - Other __________________ - Don't know - Refuse to answer | | | | | | | | | | |  |  |
| **18. Overall how confident are you that you could get advice or information about health or medical topics if you needed it?** | | | | | | | | | | | | | | | | | | | | |
| - - Completely confident | | | | | | | | | | | | | | | | | |  |  |  |
| - - Very confident | | | | | | | | | | | | | | | | | |  |  |  |
| - - Somewhat confident | | | | | | | | | | | | | | | | | |  |  |  |
| - - A little confident | | | | | | | | | | | | | | | | | |  |  |  |
| - - Not confident at all | | | | | | | | | | | | | | | | | |  |  |  |
| - - Don't know | | | | | | | | | | | | | | | | | |  |  |  |
| - - Refuse to answer | | | | | | | | | | | | | | | | | |  |  |  |
| **19. What is the first hospital that comes to mind when you think about cancer care?** | | | | | | | | | | | | | | | | | | | | |
| ____________________________________________________________________________ | | | | | | | | | | | | | | | | | | | | |
| **20. Before this survey, have you ever heard of the University of California Davis, and of its Comprehensive Cancer Center / Cancer Institute?** | | | | | | | | | | | | | | | | | | | | |
| - Yes | | | - No | | | | | | - Don't know | | | | - Refuse to answer | | |  |  |  |  |  |
| **21. Is there a specific social media site you like to go to for health or medical information?** | | | | | | | | | | | | | | | | | | | | |
| - Yes | | | | - No | | | | | | - Don't know | | | | - Refuse to answer | | | |  |  |  |
| **22. If you answered yes, what social media site do you go to for health or medical information?** | | | | | | | | | | | | | | | | | | | | |
| - Facebook - Twitter - Instagram - LinkedIn | | | | | | | | | | - Pinterest - Snapchat - Other: ___________ - Don’t know - Refuse to answer | | | | | | | | | | |
| **23. Are you 49 years of age or older?** | | | | | | | | | | | | | | | | | | | | |
| - - Yes | | | | | | | | | - No (skip to #28) | | | | | | | | | | | |
| ***24. The next questions are about colorectal cancer screening.*** | | | | | | | | | | | | | | | | | | | | |
| **A Colonoscopy is an exam in which a tube is inserted in the rectum to view the colon for signs of cancer or other health problems. Have you ever had this exam?** | | | | | | | | | | | | | | | | | | | | |
| - Yes | | | | | | - No | | | | - Don't know | | | | - Refuse to answer | | | | | |  |
| **25. If you answered yes, when was the last time you had a colonoscopy?** | | | | | | | | | | | | | | | | | | | | |
| - - Within the past 10 years | | | | | | | | | | | | | | | | | | | | |
| - - More than 10 or more years ago | | | | | | | | | | | | | | | | | | | | |
| - - Don't know/not sure | | | | | | | | | | | | | | | | | | | | |
| - - Refuse to answer | | | | | | | | | | | | | | | | | | | | |
| **26. A blood stool test is a test that may use a special kit at home to determine whether the stool contains blood (like Fecal Occult Blood Test (FOBT), a Fecal Immunochemical Test (FIT) or Cologuard). Have you ever had this test using a home kit?** | | | | | | | | | | | | | | | | | | | | |
| - Yes | | | | | - No | | | | | | - Don't know | | | - Refuse to answer | | | | | |  |
| **27. If you answered yes, how long has it been since you had you last blood stool test using a home kit?** | | | | | | | | | | | | | | | | | | | | |
| - - Within the past year (anytime less than 12 months ago) | | | | | | | | | | | | | | | | | | | | |
| - - Within the past 2 years (1 year but less than 2 years ago) | | | | | | | | | | | | | | | | | | | | |
| - - Within the past 3 years (2 years but less than 3 years ago) | | | | | | | | | | | | | | | | | | | | |
| - - Within the past 5 years (3 years but less than 5 years ago) | | | | | | | | | | | | | | | | | | | | |
| - - 5 or more years ago | | | | | | | | | | | | | | | | | | | | |
| - - Don't know/not sure | | | | | | | | | | | | | | | | | | | | |
| - - Refuse to answer | | | | | | | | | | | | | | | | | | | | |
| **28. The next questions are about cervical cancer. *(If you are not a female, skip to question #32)***  **A pap exam is a procedure to test for cervical cancer in women. A pap smear involves collecting cells from your cervix-the lower, narrow end of your uterus that’s at the top of your vagina. Have you ever had a pap smear*?*** | | | | | | | | | | | | | | | | | | | | |
| - - Yes | | | | | | - No | | | | - Don't know/ not sure | | | | - Refuse to answer | | | | | |  |

| **29. If yes, how long has it been since you had your last pap smear?** | | | | |
| --- | --- | --- | --- | --- |
| - Within the past year (anytime less than 12 months ago) | | | | |
| - Within the past 2 years (1 year but less than 2 years ago) | | | | |
| - Within the past 3 years (2 years but less than 3 years ago) | | | | |
| - Within the past 5 years (3 years but less than 5 years ago) | | | | |
| - 5 or more years ago | | | | |
| - Don't know/not sure | | | | |
| - Refuse to answer | | | | |
| **30. The next questions are about breast cancer. A mammogram is an x-ray of each breast to look for breast cancer. Have you ever had a mammogram?** | | | | |
| - - Yes | - No | - Don't know/ not sure | - Refuse to answer |  |

| **31. If yes, how long has it been since you had your last mammogram?** | | | | | | | | | |
| --- | --- | --- | --- | --- | --- | --- | --- | --- | --- |
| - Within the past year (anytime less than 12 months ago) | | | | | | | | | |
| - Within the past 2 years (1 year but less than 2 years ago) | | | | | | | | | |
| - Within the past 3 years (2 years but less than 3 years ago) | | | | | | | | | |
| - Within the past 5 years (3 years but less than 5 years ago) | | | | | | | | | |
| - 5 or more years ago | | | | | | | | | |
| - Don't know/not sure | | | | | | | | | |
| - Refuse to answer | | | | | | | | | |
| **32. The next question is intended for parents of children ages 9-17. Do you have children of age of 9-17?** | | | | | | | | | |
| - - - Yes | | | - - No (skip to #34) | | | | | | |
|  | | | | | | | | | |
| **33. Human Papillomavirus (HPV) vaccine is given to prevent HPV related cancers and genital warts. The HPV vaccines available are called Cervarix, Gardasil or Gardasil 9. It is given in 2 or 3 separate doses over a 6-month period. Has your child ever received one or more doses of the HPV vaccine?** | | | | | | | | | |
| - - Yes | - - No | | | | - Don't know | - Refuse to answer | |  |  |
| **34. Hepatitis B Vaccine is given in three separate doses and has been recommended for all newborn infants since 1991. In 1995, it was recommended that adolescents be given the vaccine. Persons who may be exposed to other people's blood, such as health care workers, also may have received the vaccine. Have you ever received the 3 dose series of the Hepatitis B Vaccine?** | | | | | | | | | |
| - - Yes at least 3 doses | | | | | | | | | |
| - - Yes, less than 3 doses | | | | | | | | | |
| - - No doses | | | | | | | | | |
| - - Don't know | | | | | | | | | |
| - - Refuse to answer | | | | | | | | | |
| **35. Have you smoked at least 100 cigarettes in your entire life?** | | | | | | | | | |
| - - Yes | | - No | | - Don't know | | | - Refuse to answer | |  |

| **36. If yes, do you now smoke cigarettes…** |
| --- |
| - - Every day |
| - - Some days |
| - - Not at all |
| - - Don't know |
| - - Refuse to answer |

**37. Considering all types of alcoholic beverages, how many times during the past 30 days did you have x [men = 5, women = 4] or more drinks on an occasion?**

- - Number of times: ______
  - None
  - Don’t know/ not sure
  - Refuse to answer

| **38. Now think about the foods you ate during the past month that is, the past 30 days, including meals and snacks. During the past month, how many times did you eat fruit? Do not count juices. You can tell us per day, per week, or month.** | |
| --- | --- |
| - __________times per day | |
| - __________times week | |
| - __________times month | |
| - Don't know | |
| - Refuse to answer | |
| **39. Now think about the foods you ate during the past month that is, the past 30 days, including meals and snacks. During the past month, how many times did you eat any other vegetables like green salad, green beans or potatoes? Do not include fried potatoes.** | |

- __________times per day
- __________times week
- __________times month
- Don't know
- Refuse to answer

**40. The next question is about your overall exercise. Exercise can include walking, housekeeping, construction, hard manual labor, field work, jogging, weights, a sport or playing with your kids. It can be done on the job, around the house, just for fun or as a work-out. In the past 7 days, on how many days did you exercise for at least 20 minutes at a time?**

- - ______ days per week
  - Don't know
  - Refuse to answer

**41. How tall are you without shoes?**

- - ________feet/________inches
  - ________meters /________centimeters
  - Don’t know
  - Refuse to answer

**42. How much do you weight without shoes?**

- - __________pounds
  - __________kilograms
  - Don’t know
  - Refuse to answer

**43. About how long has it been since you last saw a doctor or medical provider for a routine check-up? [if needed: a routine check-up is a visit not for an illness or problem. This visit may include questions about health behaviors such as smoking.]**

- - One year ago, or less
  - More than 1 up to 2 years ago
  - More than 2 up to 5 years ago
  - More than 5 years ago
  - Never
  - Don't know
  - Refuse to answer

**44. Would you say that in general your health is excellent, very good, good, fair, or poor?**

- - Excellent
  - Very good
  - Good
  - Fair
  - Poor
  - Don’t know
  - Refuse to answer

**45. Is there a place that you usually go to when you are sick or need advice about your health?**

- - Yes
  - There is no place
  - There is more than one place
  - Don't know/not sure
  - Refuse to answer

**46. If yes, what kind of place do you go most often?**

- Clinic or health center
- Doctor's office or HMO
- Hospital emergency room
- Hospital outpatient department
- Some other place
- Doesn't go to one place most often
- Don't know/not sure
- Refuse to answer

**47. During the past 12 months, did you delay or not get any other medical care you felt you needed—such as seeing a doctor, a specialist, or other health professional?**

| - - Yes | - No | - Don't know | | - Refuse to answer |  |  |  |
| --- | --- | --- | --- | --- | --- | --- | --- |
| **48. If yes, what was the one main reason why you delayed getting the care you felt you needed?** | | | | | | | |
| - Couldn’t get appointment - My insurance not accepted - Insurance did not cover - Language problems - Transportation problems - Hours not convenient - No child care for children at home | | | | - - Forgot or lost referral   - I didn’t have time   - Couldn’t afford/cost too much   - No insurance   - Other (specify: ____________)   - Don't know   - Refuse to answer | | |  |

**49. Have you ever been told by a doctor or other health professional that you had cancer or a malignancy of any kind?**

| - Yes | - No (skip to #52) | - Don't know | - Refuse to answer |
| --- | --- | --- | --- |

**50. If yes, what type of cancer was it?**

**_____________________________________________________________________**

**51. Since your diagnosis, has anyone discussed with you whether you would like to take part in cancer research? If yes, did you participate and in what?**

- - Clinical trials
  - Biospecimen donation
  - Other: _____________________________________
  - Don’t Know
  - Refuse to answer

| **52. What do you think are the most important cancer health problems in your community?** | | | | | | | | |
| --- | --- | --- | --- | --- | --- | --- | --- | --- |
| **_____________________________________________________________________________**  **_____________________________________________________________________________** | | | | | | | | |
| **53. Do you have a family history of cancer?** | | | | | | | | |
| - - Yes | | - No (skip to #54) | | | - Don't know | | - Refuse to answer |  |
| **If yes, what type of cancers have affected your family?** | | | | | | | | |
| **_____________________________________________________________________** | | | | | | | | |
| **54. What media do you use to learn about advances in health, medicine, cancer care?** | | | | | | | | |
| - - Social media | | | | | | | | |
| - - Email | | | | | | | | |
| - - Mail | | | | | | | | |
| - - Newspaper | | | | | | | | |
| - - Radio | | | | | | | | |
| - - TV | | | | | | | | |
| - - Don’t Know | | | | | | | | |
| - - Refuse to answer | | | | | | | | |
| **55. If you chose TV, in what language do you see the programs?** | | | | | | | | |
| - English | | | | | | | | |
| - Spanish | | | | | | | | |
| - Other______________ | | | | | | | | |
| - Don’t know | | | | | | | | |
| - Refuse to answer | | | | | | | | |
| **56. What Country were you born in?** | | | | | | | | |
| - - Country: _____________ | | | | | | | | |
| - - Don’t know | | | | | | | | |
| - - Refuse to answer | | | | | | | | |
| **57. How many years have you been living in the United States?** | | | | | | | | |
| - - Years: ____________ | | | | | | | | |
| - - Don’t Know | | | | | | | | |
| - - Refuse to answer | | | | | | | | |
| **58. Do you travel to Mexico or another country to see a doctor?** | | | | | | | | |
| - - Yes | | | - No | - Don't know | | | - Refuse to answer | |
| **59. Have you ever been diagnosed with one of the following? You can select more than one.** | | | | | | | | |
| - - Helicobacter Pylori Infection | | | | | | | | |
| - - Gastritis | | | | | | | | |
| - - Gastric Ulcer | | | | | | | | |
| - - Gastro-Esophageal Reflux (GERD) | | | | | | | | |
| - - Any other stomach problem: _____________________ | | | | | | | | |
| - - No (skip to #62) | | | | | | | | |
| - - Don’t Know | | | | | | | | |
| - - Refuse to answer | | | | | | | | |
| **60. Did you receive treatment for the condition above? (Helicobacter pylori, Gastric Ulcer, Gastritis, Gastro-Esophageal Reflux)** | | | | | | | | |
| - - Yes | - No (skip to #62) | | | | | - Don't know | - Refuse to answer | |
| **61. If yes, what was the treatment received for the condition?** | | | | | | | | |
|  | | | | | | | | |
| **62. Have you ever been diagnosed with one of the following?** | | | | | | | | |
| - - Liver Cirrhosis | | | | | | | | |
| - - Hepatitis B | | | | | | | | |
| - - Hepatitis C | | | | | | | | |
| - - Non-Alcoholic Fatty Liver (NAFL) | | | | | | | | |
| - - Non-Alcoholic Steatohepatitis (NASH) | | | | | | | | |
| - - Other Liver Disease: ____________________ | | | | | | | | |
| - - No (skip to the end) | | | | | | | | |
| - - Don’t Know | | | | | | | | |
| - - Refuse to answer   *Continue to the end🡪* | | | | | | | | |
| **63. Did you receive treatment for the condition above? (Liver Cirrhosis, Hepatitis B, Hepatitis C, Non-Alcoholic Fatty Liver or Non-Alcoholic Steatohepatitis)** | | | | | | | | |
| - - Yes | | | - No | | | - Don't know | - Refuse to answer | |
| **64. If yes, what was the treatment?** | | | | | | | | |
|  | | | | | | | | |
|  | | | | | | | | |
| **Please feel free to leave a comment on the survey, cancer, your community needs etc** | | | | | | | | |

______________________________________________________________________________
